# A new mesophotic goby, *Palatogobius incendius* (Teleostei: Gobiidae), and the first record of invasive lionfish preying on undescribed biodiversity

**DOI:** 10.1371/journal.pone.0177179

**Published:** 2017-05-25

**Authors:** Luke Tornabene, Carole C. Baldwin

**Affiliations:** Department of Vertebrate Zoology, National Museum of Natural History, Smithsonian Institution, Washington DC, United States of America; University of California Santa Cruz, UNITED STATES

## Abstract

A new species of deep-reef fish in the goby genus *Palatogobius* is described from recent submersible collections off Curaçao and Dominica. Video footage of schools of this species reveal predation by the invasive Indo-Pacific lionfish (*Pterois* spp.), the first record of undescribed fauna potentially being eaten by lionfish outside of its native range. We present molecular phylogenetic data for all valid species of *Palatogobius* and related genera, as well as a taxonomic key to the species of *Palatogobius* and a generic key to *Palatogobius* and related genera in the western Atlantic. Lastly, we discuss ecological and behavioral aspects of some deep-reef fishes in light of potential threats from invasive lionfish.

## Introduction

Determining the total number of extant species on Earth is a challenging objective for biologists. Recent estimates suggest there are either 5±3 million [1] or 8.7±1.3 million [2] species, millions of which have yet to be discovered and described. Estimates of contemporary extinction suggest that the Earth is currently experiencing a 6^th^ period of mass extinction driven entirely by anthropogenic causes [3–6], and there is growing concern that a large percentage of undescribed species will go extinct before they can be described [1, 7]. There is special impetus to formally describe taxa that may have elevated extinction risks associated with the specific region and ecosystem that they inhabit (e.g., Caribbean coral reefs, Amazonian rainforests).

Among the leading causes of contemporary animal extinctions are invasive species [8–13], which can negatively impact native populations via direct predation on smaller species, habitat degradation, introduction of pathogens, and competition for resources. The most prevalent and unabating invasive species currently threatening marine ecosystems are the Indo-Pacific lionfishes, *Pterois volitans* and the morphologically similar *P*. *miles* (hereafter collectively referred to as ‘lionfish’). The first records of lionfish in the western Atlantic date either to 1985 [14] or to the early 1990’s [15], and lionfish subsequently spread throughout the Caribbean, in the western Atlantic from Brazil to Rhode Island, and into the Mediterranean Sea. They are now abundant on coral reefs and in other coastal habitats throughout most of their introduced range [16–21]. Lionfish are extremely effective predators of small reef fishes, including critically endangered taxa [22], and have caused dramatic declines in native reef fish biomass and recruitment [23–26].

The success of the lionfish invasion can be attributed to several factors, including high reproductive output and rapid growth [27, 28], a lack of major predators (but see [29, 30]), and their highly effective feeding strategies [25, 28, 31, 32]. Lionfish are especially adept at preying on species that are small, shallow bodied, and hover over the substrate [33]. These characteristics are present in many species of fishes in the family Gobiidae, commonly known as gobies. Gobies are the most diverse and numerically abundant family of fishes on coral reefs, and contribute substantially to reef trophodynamics [34]. Gobies collectively make up a large percentage of the overall diet of invasive lionfish, and species of gobies are frequently the most abundant fishes found in lionfish guts [14, 22, 23, 24, 28, 35, 36]. Unlike many other reef fishes that can grow large enough to avoid predation as adults, gobies are vulnerable to predation by lionfish both as juveniles and adults. As a result, lionfish have led to local declines in biomass and recruitment in some Caribbean gobies, and several species are now listed as ‘near threatened,’ ‘vulnerable,’ or ‘endangered’ by the IUCN Red List of Endangered Species, due in part to potential threats by lionfish [13].

Lionfish are primarily crepuscular hunters, and hunt more actively on overcast days with less light and at greater depths [32, 35]. In the western Atlantic lionfish are tolerant of, and can even thrive at, the cooler temperatures of mesophotic and deeper reefs, where they have become locally abundant at many localities [19, 37–40, Baldwin et al. unpublished data]. This is especially concerning for native deep-reef fishes that occur from 50–300 m, where reduced light conditions may make them more susceptible prey for actively hunting lionfishes. In addition, the taxonomic composition of mesophotic fish communities differs substantially from that of shallow reefs, and is made up primarily of a unique ‘deep-reef’ fauna that includes many undescribed species [41–48]. Thus, deep-reef fishes in the Caribbean represent an entire community of poorly known or undescribed species that may be negatively affected by invasive lionfish. To date little is known about the lionfish predation on deep-reef fish assemblages.

In recent years the Smithsonian’s Deep Reef Observation Project (DROP) has contributed a significant body of information on the taxonomic makeup of deep-reef fish communities in the Caribbean. Through the use of Substation Curaçao’s (http://www.substation-Cuaracao.com) manned submersible, the *Curasub*, DROP researchers have described many new species of deep-reef fishes, including several that may be susceptible to lionfish predation due to their body size, shape, and behavior. These include species of basslets in the genus *Lipogramma* [49], seabasses in the genus *Liopropoma* [50, 51], and gobies from several genera [52–55]. Lionfish are also frequently observed on *Curasub* dives down to 247 m, often in close proximity to rare or undescribed native deep-reef fishes. During a submersible dive off the west coast of Curacao in 2015, DROP scientists observed the first instance of an undescribed species of reef fish being preyed upon by lionfish. Here we document this event with video footage and provide a taxonomic description of the species, *Palatogobius incendius* n. sp. The description includes information on ontogenetic changes in coloration and is accompanied by molecular phylogenetic data from mitochondrial cytochrome c oxidase I (COI) and combined nuclear and mitochondrial data from Tornabene et al. [55]. Lastly, we provide a key to the species of *Palatogobius*, and discuss the ecology of the new species and that of other deep-reef fishes that may be targeted by invasive lionfish.

## Materials and methods

The new species of *Palatogobius* was observed and collected during several submersible dives off the coasts of Curaçao and Dominica from 2013 to 2016. Observations of gobies and the lionfish predation event were recorded from the *Curasub* submersible by a high-definition video camera mounted on the front of the sub. Digital photographs of gobies were also taken from a Nikon DSLR camera in an underwater housing mounted on one of the sub’s hydraulic arms.

Specimens were collected by the *Curasub* using the two hydraulic arms, one equipped with a quinaldine-ejection system to anesthetize fishes, and the second equipped with a suction hose to collect immobilized individuals. Captured specimens were stored in a vented acrylic container for transport to the surface. In the lab, specimens were photographed, tissue sampled and preserved.

Methods for counts and measurements follow Böhlke & Robins [56] as modified by Van Tassell et al. [52], who, like many authors, differentiate the unsegmented spine from the segmented rays of the second dorsal, anal, and pelvic fins using the Roman numeral ‘I’ for the spine followed by Arabic numbers for the soft rays. Counts for the holotype are given first, followed in parentheses by the range of the type series if different than the holotype. Measurements for the holotype are given first, followed by the average and range in parentheses. Dorsal pterygiophore formula is that of Birdsong et al. [57] and cephalic-canal and pore terminology follows Akihito et al. [58]. Institutional acronyms follow Sabaj Pérez [59]. DNA extraction and COI sequencing follow Weigt et al. [60]. Phylogeny was inferred using Bayesian inference in the program MrBayes ver. 3.2 [61], using two Metropolis-coupled Markov Chain Monte Carlo (MCMC) runs, each with four chains. The analysis was run for 10 million generations, sampling trees and parameters every 1000 generations. Burn-in, convergence and mixing were assessed using Tracer [62] and by visually inspecting consensus trees from both runs.

### Nomenclatural acts

The electronic edition of this article conforms to the requirements of the amended International Code of Zoological Nomenclature, and hence the new names contained herein are available under that Code from the electronic edition of this article. This published work and the nomenclatural acts it contains have been registered in ZooBank, the online registration system for the ICZN. The ZooBank LSIDs (Life Science Identifiers) can be resolved and the associated information viewed through any standard web browser by appending the LSID to the prefix “http://zoobank.org/”. The LSID for this publication is: urn:lsid:zoobank.org:pub: B36B2485-E2E1-4121-AB7E-F9F54E29F154. The electronic edition of this work was published in a journal with an ISSN, and has been archived and is available from the following digital repositories: PubMed Central, LOCKSS.

### Ethics statement

Specimens were collected under the auspices of the Curacao Sea Aquarium with permission of owner Adrian Schrier. This study was conducted under Smithsonian Animal Care and Use Committee (ACUC) approval to C. C. Baldwin (ACUC #2011–07). Guidelines for field activities with wild fishes, established by the American Society of Ichthyologists and Herpetologists (http://www.asih.org/files/fish%20guidelines.doc) were followed for all field collections, including euthanasia with tricaine methane sulfate (MS-222). Field studies involved no endangered or protected species.

## Results

### *Palatogobius incendius* Tornabene, D. Ross Robertson & Baldwin, sp. nov.

**Fig 1:**
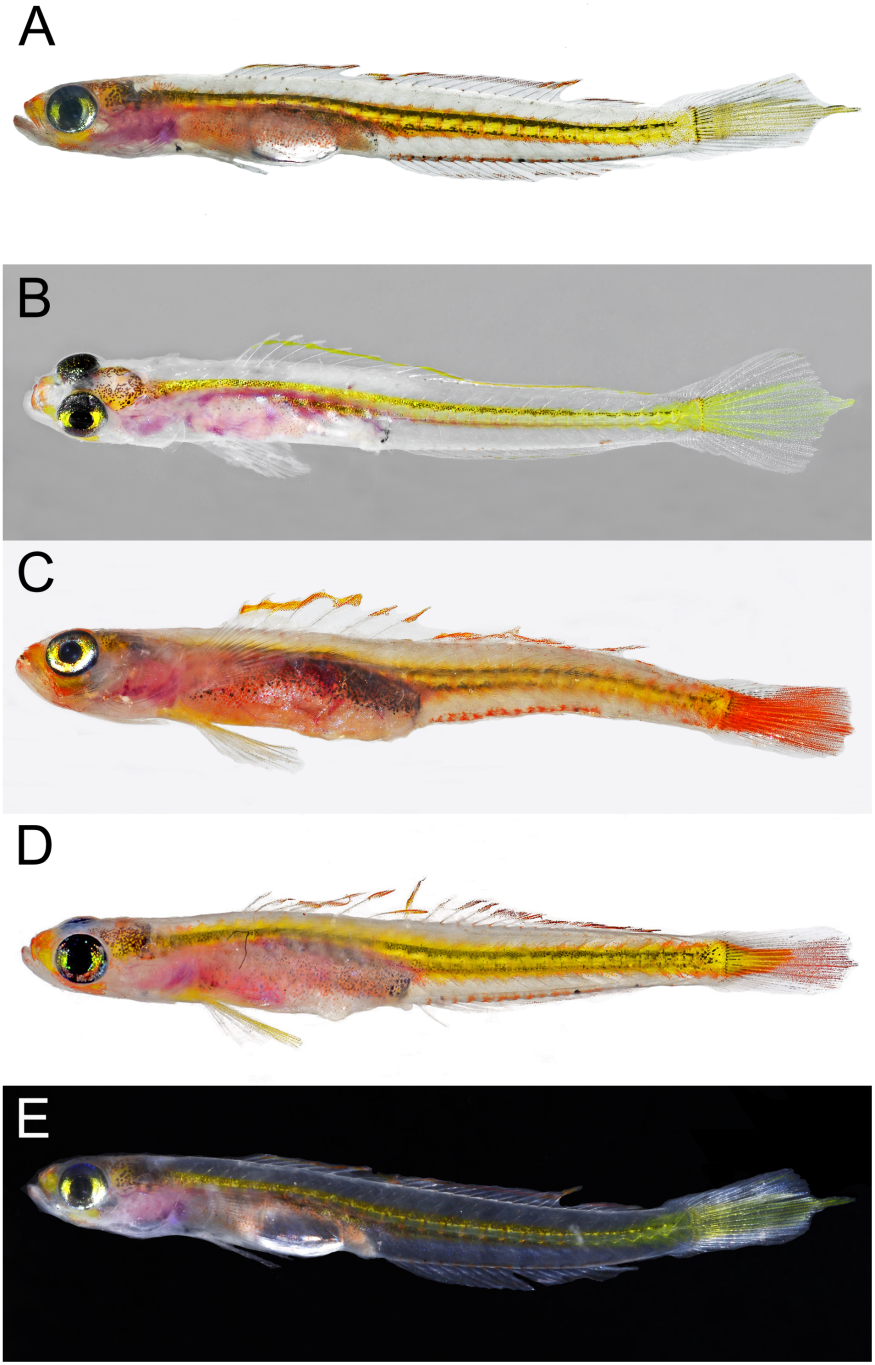
*Palatogobius incendius*, prior to preservation. A) USNM 436470, tissue CUR15135, 19 mm SL, Curacao; B) USNM 436483, tissue CUR15148, 21 mm SL, Curacao; C) USNM 431354, tissue CUR14029, 22 mm SL, Curacao; D) USNM 435318, 18.1 mm, Curacao. Photos by Carole C. Baldwin.

**Fig 2:**
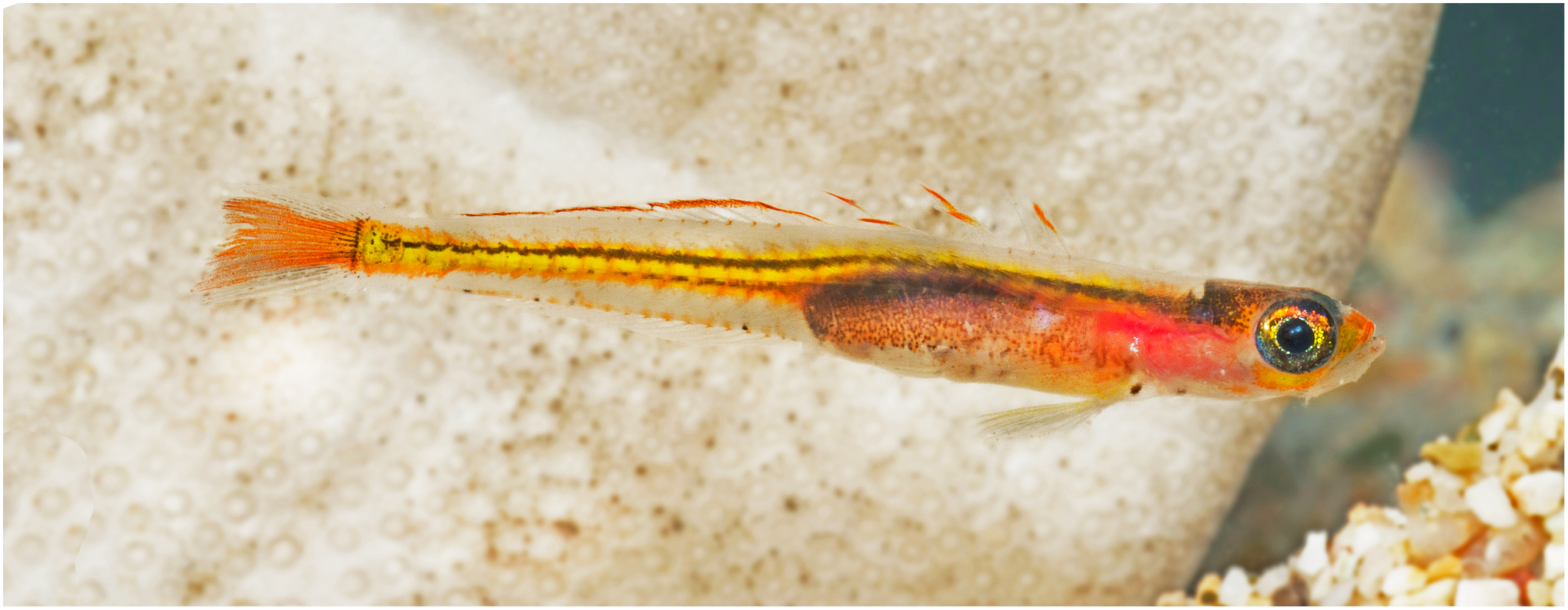
*Palatogobius incendius*, live in aquarium, USNM 415430, 18.3 mm SL, Curacao. Photo by Barry Brown.

**Fig 3:**
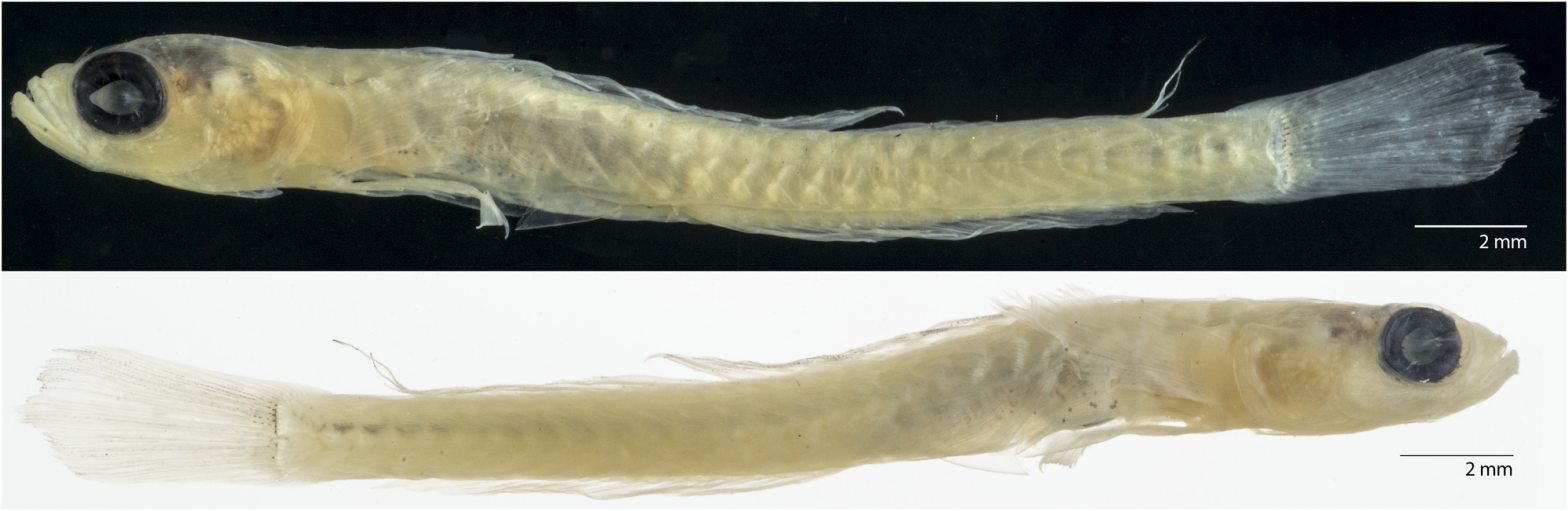
*Palatogobius incendius*, preserved holotype, USNM 410996, 22.2 mm SL, Curacao. Photos by Sandra Raredon.

**Fig 4:**
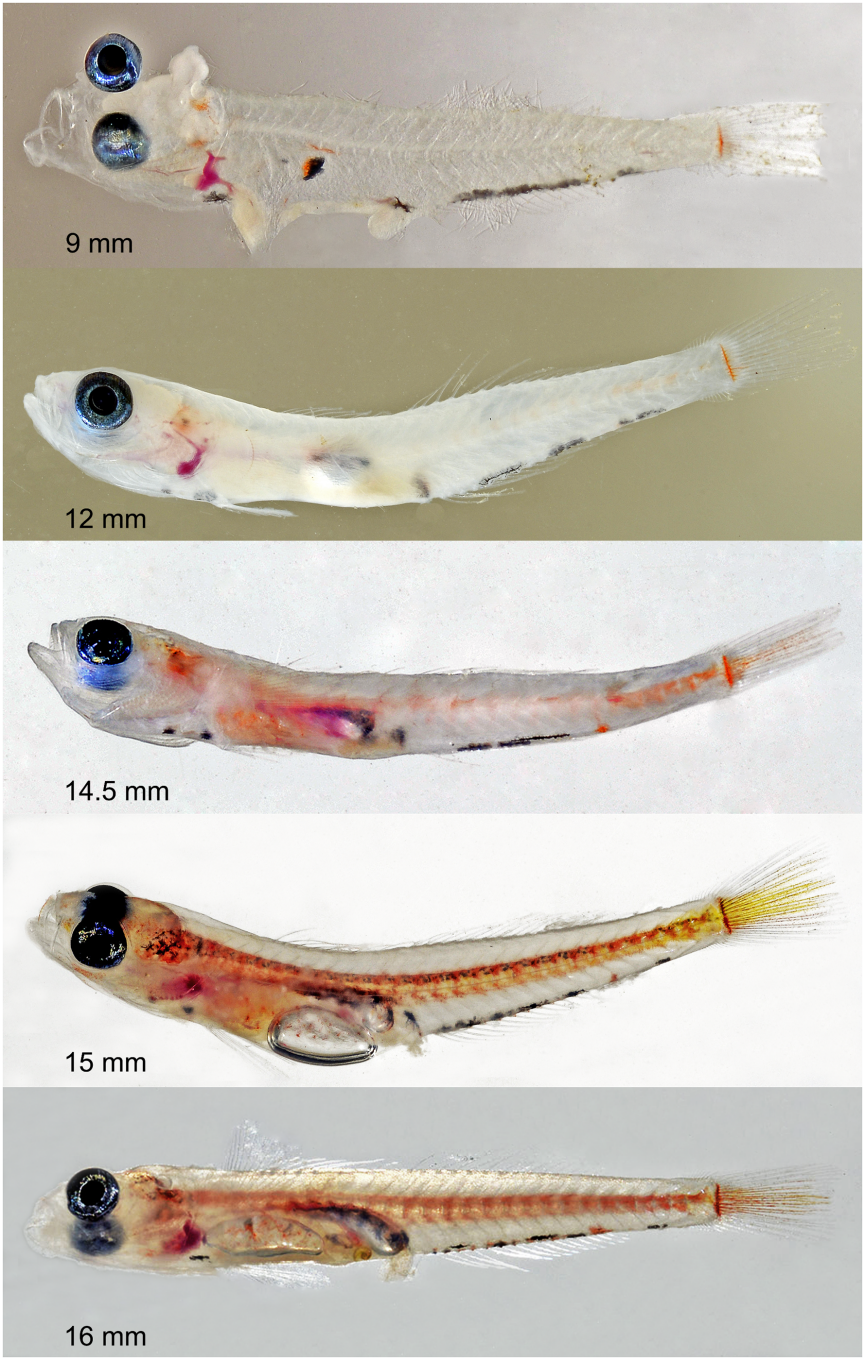
*Palatogobius incendius* larvae. Sizes are in SL. From top to bottom: no voucher, tissue DOM16044, Dominica; USNM 438696, tissue DOM16045, Dominica; no voucher, tissue CUR15016, Curacao; USNM 434800, tissue CUR15120, Curacao; USNM 434801, tissue CUR15121, Curacao. Photos by Carole C. Baldwin.

**Fig 5:**
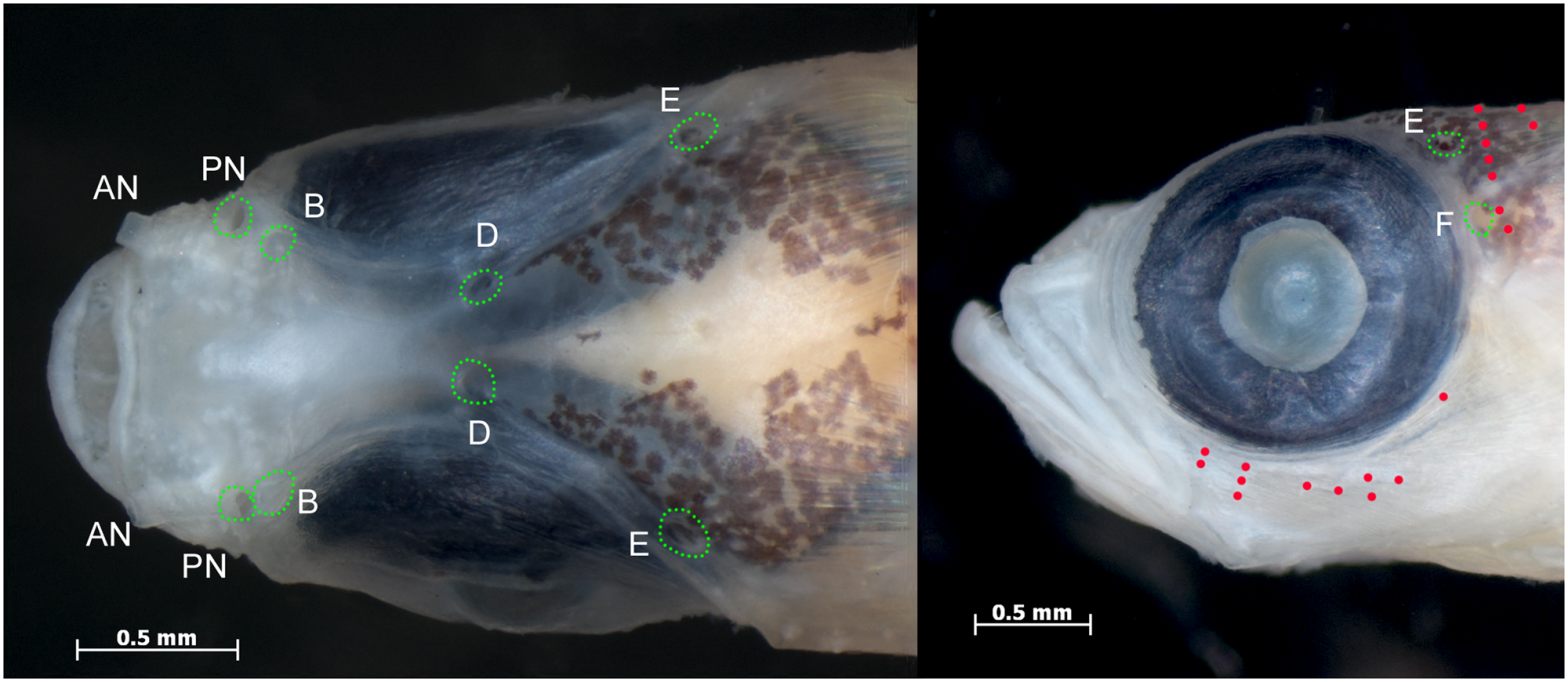
Position of sensory papillae (red) and head pores (green) in *Palatogobius incendius*, USNM 415430. AN—anterior nare, PN—posterior nare. Photograph by G. David Johnson.

**Fig 6:**
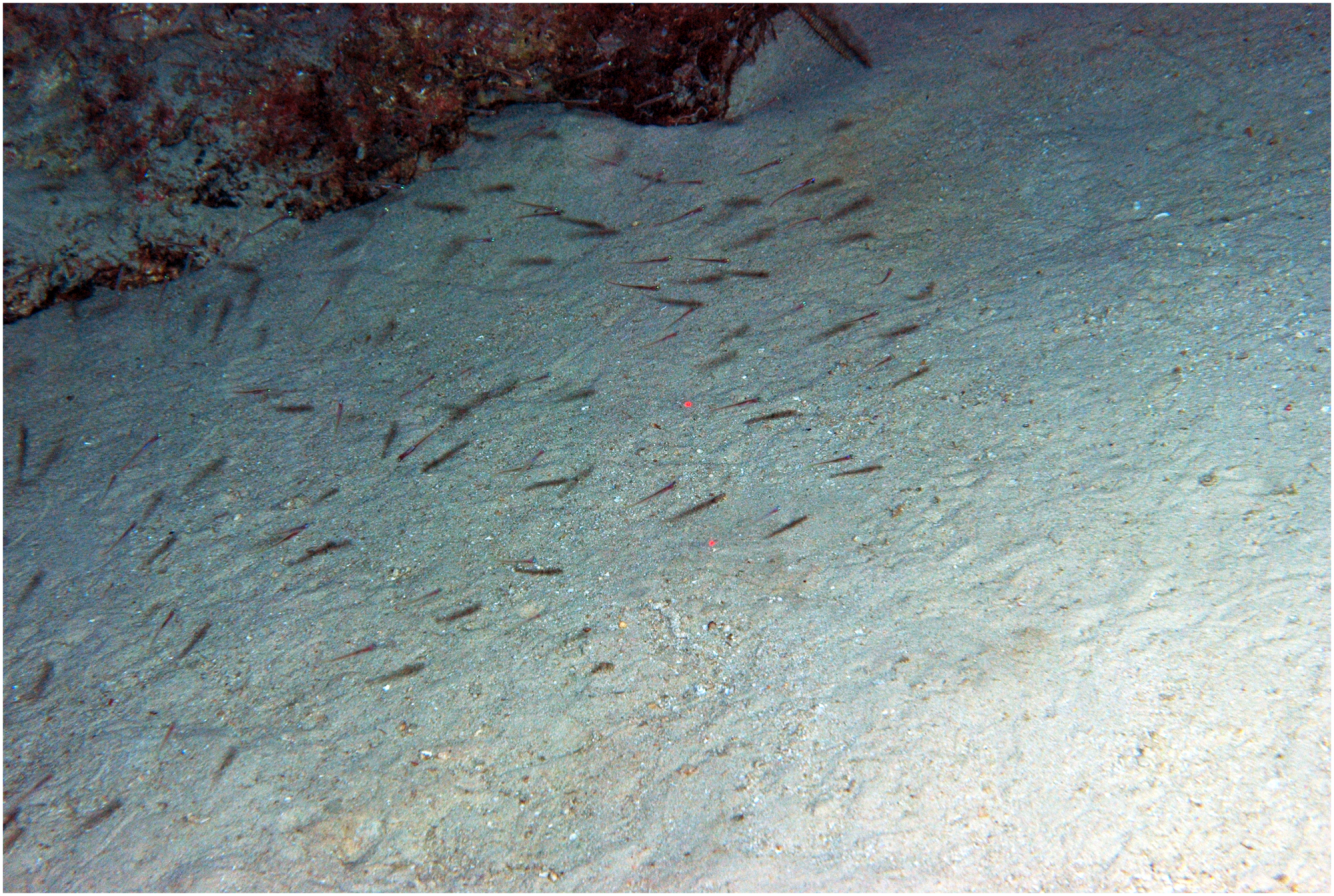
School of *Palatogobius incendius* at type locality, sta. CURASUB15-30, 152 m depth, Curacao.

urn:lsid:zoobank.org:pub: B36B2485-E2E1-4121-AB7E-F9F54E29F154, Ember Goby, Gobio de Brasas (Spanish), Figs 1–6.

#### Holotype

USNM 410996, male, 22.2 mm SL, sta. CURASUB15-30, southern Caribbean, Curaçao, leeward side of NW coast, Playa Jeremi, 12.328 N, 69.157 W, 152 m depth, 1 October 2015, Carole Baldwin, Bruce Brandt, Ross Robertson, Luke Tornabene.

#### Paratypes

Curaçao: USNM 435316, female, 17.63 mm SL, sta. CURASUB15-05, east of Substation downline, 12.083 N, 68.8991 W, 128 m depth, 10 February 2015, Carole Baldwin, Ross Robertson, Cristina Castillo, Barbara van Bebber; USNM 435317, male, 16.2 mm SL, cleared and stained, collected with USNM 435316; USNM 435315, female, 19.4 mm S, sta. CURASUB15-06, east of Substation downline, 12.083 N, 68.8991 W, 119 m depth, 11 February 2015, Carole Baldwin, Ross Robertson, Bruce Brandt, Adrian Schrier; USNM 435335, cleared and stained, tissue CUR15014, collected with USNM 435316; USNM 434800, juvenile, 12.2 mm SL, tissue CUR15020, sta. CURASUB15-23, east of Substation downline, 12.083 N, 68.8991 W, 120–122 m depth, 23 September 2015, Carole Baldwin, Tico Christiaan, Bruce Brandt; USNM 431308, juvenile, 13.1 mm SL, collected with USNM 434800; USNM 439126, 2 juveniles, cleared and stained, collected with USNM 434800; USNM 434801, head only, tissue CUR15021, collected with USNM 434800; USNM 436481, cleared and stained, tissue CUR15146, collected with holotype; USNM 436483, cleared and stained, tissue CUR15148, collected with holotype; USNM 432007, female, 21.5 mm SL, collected with holotype; USNM 436470, 19 mm SL, tissue CUR15135, sta. CURASUB15-26, Playa Forti, Westpoint, 12.368 N, 69.155 W, 125–127 m depth, Carole Baldwin, Barbara Van Bebber, Ross Robertson, Luke Tornabene; USNM 415430, female, 18.3 mm, collected with USNM 436470; USNM 435318, female, 18.1 mm SL, sta. CURASUB15-04, east of Substation downline, 12.083 N, 68.8991 W, 119 m depth, 9 February 2015, Carole Baldwin, Bruce Brandt, Cristina Castillo, Ross Robertson;

Dominica: USNM 438695, juvenile, 9 mm SL, tissue DOM16044, sta. CURASUB16-12, Prince Rupert Bay, Portsmouth, 15.557 N, 61.4709 W, 155 m depth, 8 March 2016, Carole Baldwin, Luke Tornabene, Bruce Brandt, Barrett Brooks; USNM 438696, juvenile, tissue DOM16045, collected with USNM 438695.

#### Diagnosis

Side of body with yellow/orange stripe along lateral midline, stripe continuing onto and extending entire length of caudal fin; second dorsal fin I,14–16; anal fin I,14–17; pectoral fin 18–20; no teeth on vomer; body scales absent except for occasionally 2 to 3 cycloid scales on base of caudal fin; interorbital pore C absent; interorbital pores D paired; eye diameter 7.0–9.0% SL.

#### Description

Overall body shape elongate and slender, eye large, mouth small, fins relatively short and delicate. Morphometric data given in Table 1.

**Table 1:** Morphometrics.

Sex	USNM 410996	USNM 435316	USNM 435318	USNM 415430	USNM 432007	Mean
	Holotype	Paratype	Paratype	Paratype	Paratype	
	male	female	female	female	famale	
SL	**22.2**	17.6	18.1	18.3	21.5	**19.5**
head (% SL)	**24.9**	24.4	24.3	25.4	21.4	**24.1**
eye (% SL)	**7.2**	9.0	8.3	7.9	7.0	**7.9**
eye (% HL)	**28.9**	36.7	34.1	31.2	32.6	**32.7**
jaw (% SL)	**6.8**	8.4	7.7	8.2	7.4	**7.7**
jaw (% HL)	**27.3**	97.7	31.8	32.3	34.8	**44.8**
snout (% SL)	**3.8**	4.1	4.4	3.8	4.2	**4.1**
snout (% HL)	**15.4**	17.0	18.2	15.1	19.6	**17.0**
body depth at first dorsal (% SL)	**10.8**	12.0	12.2	11.5	11.2	**11.5**
caudal peduncle depth (% SL)	**12.1**	12.0	17.7	15.3	14.0	**14.2**
caudal peduncle length (% SL)	**7.4**	7.4	6.9	6.2	6.5	**6.9**
pectoral length (% SL)	**17.5**	17.2	16.0	15.3	14.9	**16.2**
pelvic length (% SL)	**19.0**	15.9	19.3	15.8	14.9	**17.0**
caudal length (% SL)	**21.6**	18.0	17.7	17.9	17.7	18.6

Fins: counts taken from six cleared and stained specimens plus up to five additional adult specimens if counts were possible given the condition of specimens: first dorsal fin VII, no spines notably elongate; second dorsal fin I,15 (I,14–16); anal fin I,15 (I,14–17); all dorsal-fin rays and spines extremely thin, delicate, and poorly ossified; first and second dorsal fins connected by low membrane in large males, making fins appear continuous; pectoral fin 19 (18–20), extending posteriorly to beneath last spine of first dorsal fin; pelvic fin I,5, fins united by membrane to form oval disc with thin anterior frenum connecting pelvic spines, frenum of many paratypes torn or missing completely due to damage, pelvic fin extending posteriorly to vertical through origin of 4^th^ or 5^th^ dorsal spine, not reaching anus; caudal fin with 16 (15–16) segmented rays, 12 (11–12) branched rays, fin shape variable, truncate or slightly bilobed in some females, some larger males with middle three rays slightly elongate, producing a lanceolate, diamond, or spade shape.

Scales: body and head completely lacking scales except for two specimens with 2 or 3 large, embedded cycloid scales at base of caudal fin.

Head: head laterally compressed, length 22.2% SL (19.5, 17.6–22.2); eye very large, 7.2% SL (7.9, 7.0–9.0); jaw short, angled upwards approximately 20–30 degrees from horizontal, 6.8% SL (7.7, 6.8–8.4), extending posteriorly to, or falling slightly short of, vertical through anterior margin of pupil; snout short, 3.8% SL (4.1, 3.8–4.4); both jaws with two rows of narrow pointed teeth, anterior teeth in outer row greatly enlarged, recurved canines; no teeth on vomer; anterior naris a very short tube just above upper lip, posterior naris an opening with slightly raised rim.

Color in life or fresh specimens (Figs 1 and 2): head and trunk predominantly translucent; patches of bright yellow or orange on snout, side of head below eye, and on nape; nape with scattered dark melanophores, more concentrated posteriorly; iris of eye highly reflective, with an iridescent, metallic, yellow, lateral stripe, silver or metallic blue above and below; thoracic and abdominal regions pink or maroon subcutaneously, presumably from blood vessels; gas bladder visible through side of body, covered with scattered dark melanophores, reflective leucophores, and red and orange chromatophores; body with bright yellow, internal, lateral stripe along vertebral column, sometimes two narrower stripes present one above the other, yellow stripe(s) often associated with pair of narrow dark stripes made up of concentrations of melanophores; yellow lateral stripe also associated with numerous orange markings, each of which appears to be associated with myosepta; this orange pigment sometimes covering most of posterior portion of caudal peduncle and sometimes appearing as a stripe dorsally and ventrally bordering yellow stripe; yellow/orange lateral stripe broadening on base of caudal fin and continuing to tip of fin, areas of caudal fin above and below stripe translucent; dorsal and ventral midlines of body frequently with orange subcutaneous spots at base of each second-dorsal and anal-fin pterygiophore, and a few melanophores sometimes present in association with orange spots along anal-fin base; dorsal fins with narrow bright yellow or orange distal stripe, remainder of fin transparent; pectoral fin transparent; pelvic fin transparent or with yellow wash on fin base and innermost rays.

Color pattern of preserved holotype (Fig 3): When photographed against a white background, color pattern fairly uniform yellowish white. A few scattered black melanophores on side of belly, two slightly darker grey areas (a triangular patch behind the eye and an elongate blotch along mid-side above the rear half of the stomach cavity), a row of grey spots along mid-flank that become progressively darker towards the rear of the body, and partial definition of muscle blocks on the body with vague dark and light marks. These color elements also visible and more strongly defined in holotype when photographed against black background.

Larval/Juvenile coloration: An ontogenetic series from 9.0–16.0 mm SL is shown in Fig 4. Specimens of 9.0 and 12.0 mm SL with little pigment except for prominent series of melanophores along anal-fin base, orange bar at base of caudal fin, small patch of orange mixed with black internally in abdominal region, small patches of pale orange behind eye in temporal region, and black spots on ventral midline in thoracic region. Traces of lateral yellow/orange stripe characteristic of adults first apparent in 12.0-mm SL specimen along posterior portion of vertebral column, this region with considerably more orange pigment in 14.5-mm SL specimen, which also has scattered orange pigment more anteriorly along vertebral column, more orange in temporal region, orange pigment on caudal fin, a single orange spot along anal-fin base, and more orange in abdominal region. Black pigment still present along anal-fin base and in abdominal and thoracic regions. Specimens of 15.0 and 16.0 mm SL with considerably more pigment defining lateral stripe, this stripe now continuing broadly onto caudal fin, orange bar at base of caudal fin now mixed with melanophores, more orange spots appearing along base of anal fin, and orange pigment now visible on snout and cheek.

Head pores and sensory papillae (Fig 5): Sensory papillae poorly developed, and damaged on most specimens; when intact, several short transverse rows of papillae on side of head and a transverse row extending partially over nape; cephalic lateralis system consisting of pair of canals with pores B′, D, E, and F′, all paired; preopercular canal and pores absent.

Genital papillae: male papilla narrow and pointed, female papilla short and bulbous.

#### Etymology

The specific epithet *incendius* is an adjective formed from the Latin root *incendium* meaning ‘fire.’ The scientific and proposed common names refer to the bright orange, yellow and reddish-pink coloration on the body, head and fins.

#### Habitat and distribution

*Palatogobius incendius* has been collected on deep reefs from Curacao (119–128 m) and Dominica (88–168 m) and observed off Roatan, Honduras (94–201 m). The species occurs exclusively in hovering schools ranging in size from as small as 5 to 10 individuals (rare) to 50->200 individuals (Fig 6). Schools are most frequently found at the top or bottom of vertical walls off Curaçao and Dominica, but off Roatan we observed more than a dozen schools of *P*. *incendius* collectively comprising as many as 1000 individuals over a long, gradually inclining stretch of sand and small rocks from ~150–170 m depth. Over this stretch, *P*. *incendius* co-occurred with many individuals of *P*. *grandoculus*, which were closer to the bottom rather than hovering well off the bottom like *P*. *incendius*. Schools of *P*. *incendius* generally comprise individuals at multiple life stages, ranging from moderately developed larvae (~9 mm SL) to adults. Off Dominica we also observed larger swarms of minuscule fish (~5 mm TL) that could possibly be very recently recruited *P*. *incendius* larvae, given their size, abundance and depth range. Individuals in these swarms were too small to be captured, and were observed traveling only a few cm off the bottom rather than hovering in a cloud well above the substrate. These schools of post-larvae were 1–2 m wide and up to 5 m long, and moved steadily upslope at approximately 0.15 m/s, navigating laterally around obstacles in a fashion superficially similar to a wide chain of marching army ants.

### Lionfish predation event

Video of the lionfish predation event described below is viewable at https://youtu.be/YB2fRb4WVjE, and downloadable at Dryad (www.datadryad.org, doi:10.5061/dryad.s3tc6).

On 9 February 2015 a team of DROP scientists dived in the *Curasub* submersible off the west coast of Curaçao (Dive number CURASUB15-4). The sub was launched from the dock at Substation Curaçao and submerged off the outer reef slope following a navigational downline. The team descended and headed east from the downline, collecting samples along a sandy rubble slope interrupted with steep rock ledges from 64–86 m. Several specimens of *Chromis insolata*, *Lipogramma klayi* and *Liopropoma mowbrayi* were collected along this slope, and two lionfish were observed around rocky outcroppings around 66 and 76 m. At 87 m the slope transitioned to a vertical rock wall with scattered gorgonians and whip corals. At 117 m the first individuals of *Palatogobius incendius* were observed. A school of approximately 50 fish was recorded hovering off the face of the rock wall, with a lionfish hovering immediately above them on the slope. The lionfish approached the school from above with dorsal and pectoral fins expanded, and slowly corralled the group before opening its gape and striking using a powerful stroke from the caudal and pectoral fins. The lionfish continued stalking the school with dorsal- and pectoral-fin rays erected until the gobies grouped with a larger school (>100 individuals). At this point, approximately 1 minute 15 seconds after the first strike, the lionfish herded a small part of the school beneath a rocky overhang and cornered them against the wall, where it made another strike, this time scattering the entire school. Immediately following this strike the lionfish made five or six deliberate opercular movements, quickly expanding and contracting its branchial chamber in concert with opening and closing its mouth, possibly to pass captured prey into its stomach, before continuing up the slope to follow the school. A specimen of *P*. *incendius* was collected from the school (USNM 435318) before the sub team concluded the dive. We note that this individual was collected after the video recording was made, and no quinaldine was dispensed in the area prior to making the video.

## Discussion

### Phylogenetic relationships and taxonomy

**Fig 7:**
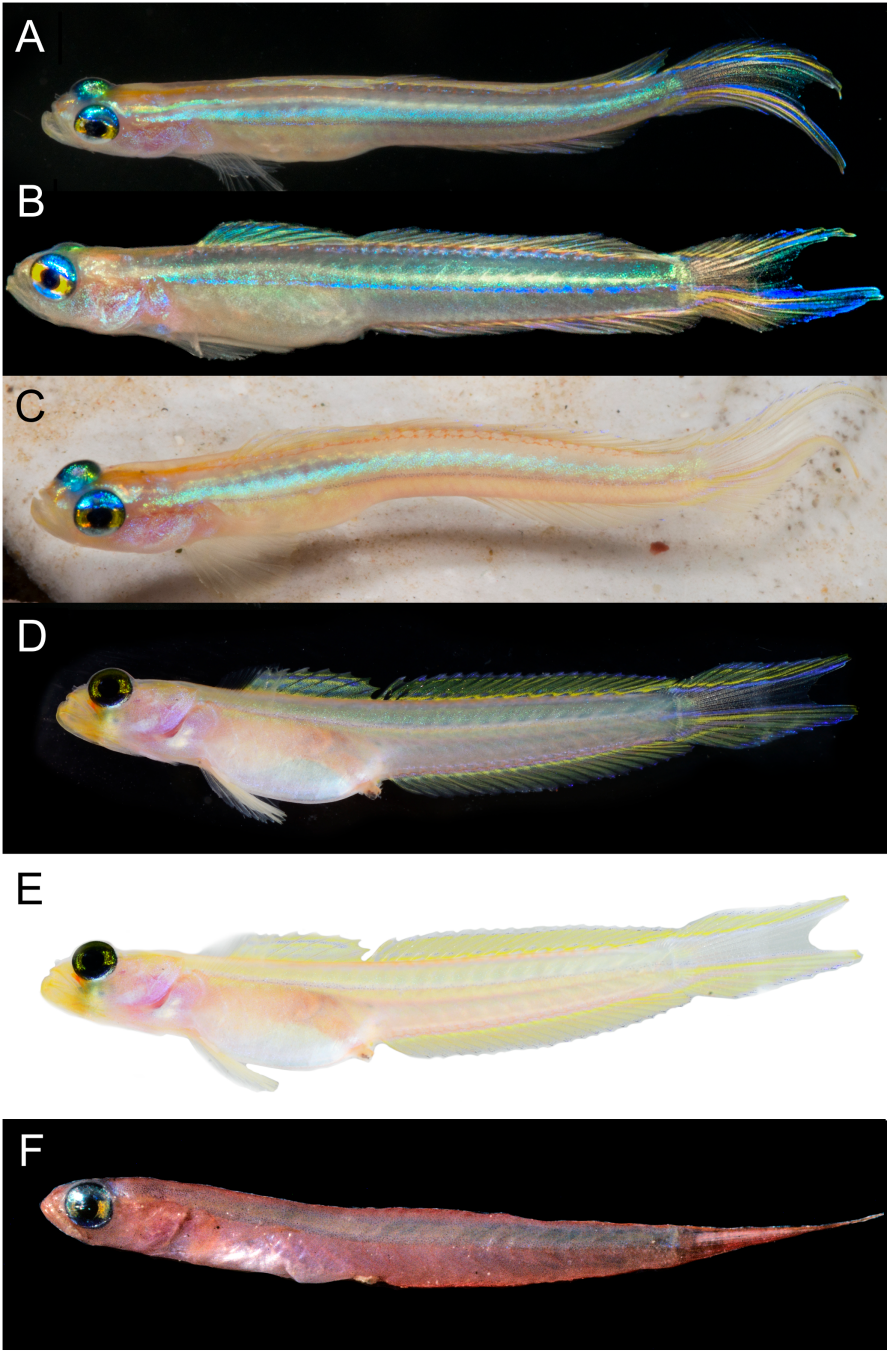
Palatogobius paradoxus. A-C) live, USNM 436469, tissue CUR15134, Curacao, photo by Barry Brown; D, E) before preservation, USNM 438754, tissue DOM16103, Curacao, photos by Carole C. Baldwin and Ross Robertson; F) *Palatogobius* cf. *paradoxus*, UF 152158, Gulf of Mexico, photo by Philip Hastings.

**Fig 8:**
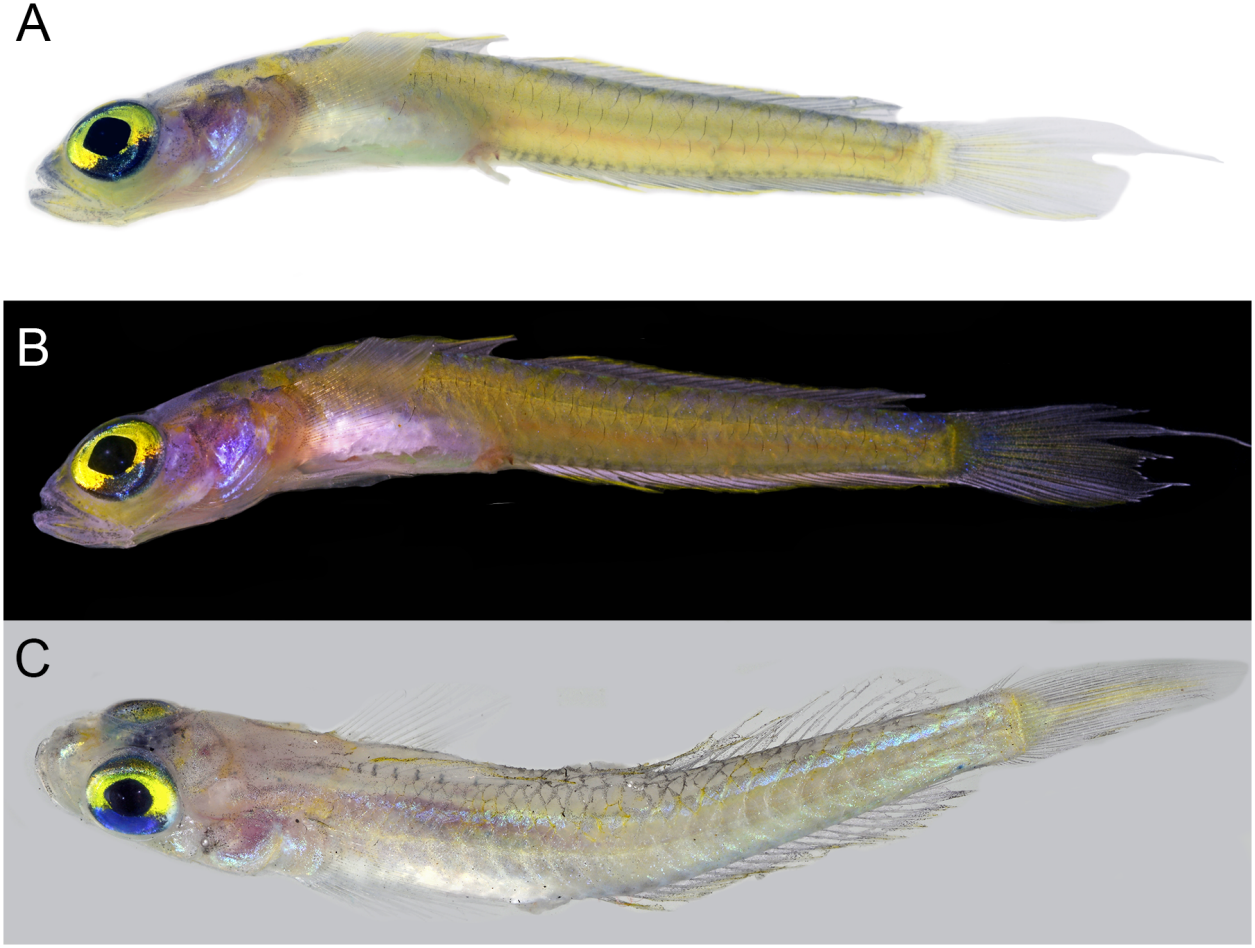
Palatogobius grandoculus. A, B) USNM 438754, tissue DOM16103, Dominica, photo by Carole C. Baldwin; C) USNM 426791, tissue CUR13284, Curacao, photo by Carole C. Baldwin and Ross Robertson.

The genus *Palatogobius* was described by Gilbert [63] and previously included two species [63–65], *P*. *paradoxus* Gilbert, 1971 (Fig 7), and *P*. *grandoculus* Greenfield, 2002 (Fig 8). Birdsong et al. [57] and Van Tassell et al. [52] supported the placement of *Palatogobius* in the *Microgobius* group of the tribe Gobiosomatini based on the presence of seven spines in the first dorsal, 1 epural, 11+16 vertebrae, two anal-fin pterygiophores anterior to the first haemal spine, a 3–221110 dorsal pterygiophore pattern, and lack of fusion of hypurals 1–2, hypurals 3–4 and the urostyle. Van Tassell et al. [52] provided a key to the genera of the *Microgobius* group and a table of putatively informative characters for the six genera in the group (*Antilligobius*, *Bollmannia*, *Parrella*, *Microgobius*, *Akko*, and *Palatogobius*). With the addition of *P*. *incendius*, several characters listed by Van Tassell et al. [52] for *Palatogobius* require amending (i.e. the presence or absence of a pelvic fraenum, range of counts for dorsal and anal rays, sensory pore pattern, lateral scale rows, presence or absence of a gas bladder). We provide an updated key to the genera of the *Microgobius* group from the Western Atlantic that accounts for these changes (see Supporting Information S1 File).

**Fig 9:**
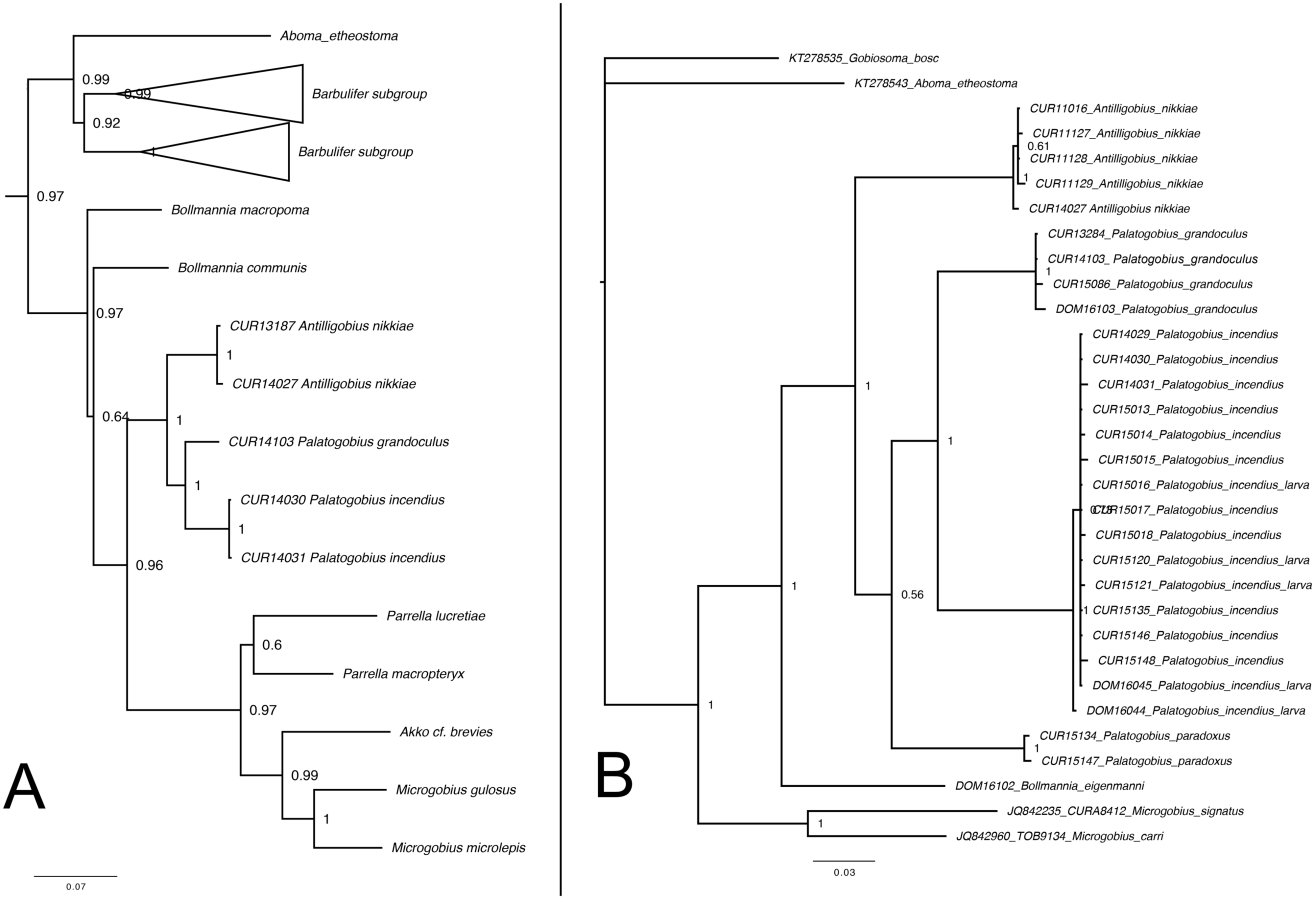
Molecular phylogenies. A) five-gene phylogeny based on data from Tornabene et al. [48]; B) phylogeny based on COI data. Support values are Bayesian posterior probabilities.

The molecular phylogeny by Tornabene et al. [48] was the first formal phylogenetic analysis to include species of *Palatogobius*. Their tree (Fig 9A) was based on four nuclear markers and mitochondrial cytochrome b, and confirmed the placement of *Palatogobius* within the *Microgobius* group of the Gobiosomatini, and a sister relationship between *Palatogobius* (*P*. *grandoculus* and *P*. *incendius*) and *Antilligobius*. We provide phylogenetic data from an additional gene, COI, and 22 additional specimens of *Palatogobius*, including all three valid species (Fig 9B). Our tree based on COI provides only weak support for the monophyly of *Palatogobius* but strong support for a sister relationship between *Palatogobius* and *Antilligobius* and for the sister relationship between *P*. *grandoculus* and *P*. *incendius*. There is no single morphological synapomorphy for the genus *Palatogobius*, but the genus can be morphologically diagnosed by a combination of characters that are variably present in other *Microgobius* group genera: (i) preopercular sensory pores M’, N and O’ absent (also in *Akko* and some *Parrella* spp.); (ii) basihyal narrow, not spatulate or bifid (also in *Akko*); (iii) metapterygoid very narrow, not wider than sympletic (also in *Antilligobius*); (iv) ventral post-cleithrum present (also in *Bollmannia* and *Antilligobius*); (v) first haemal arch expanded, considerably larger than second arch (also in *Antilligobius*). Several of these characters are losses or morphological reductions, and have likely occurred independently in several genera.

There is uncertainty in the literature regarding the diagnostic characters defining *Palatogobius paradoxus* that warrant clarification. Gilbert [63] described *P*. *paradoxus* based on the holotype (ANSP 109182) from 21 m depth off the Virgin Islands, and two other specimens from 63–71 m depth off Panama (UF 223118, formerly UMML 23118, now cleared and stained) and Venezeula (UF 226700, formerly UMML 26700). He noted several differences between the holotype and the other two specimens (caudal fin forked vs. lanceolate, vomerine teeth present vs. absent, and others), and refrained from designating them as paratypes because of these discrepancies. Gilbert [64] later described a series of *Palatogobius* from 27–39.5 m depth in the northern Gulf of Mexico (UF 152154–152158), which he also assigned to *P*. *paradoxus*. Philip Hastings photographed one of these specimens shortly after capture (Fig 7F; UF 152158). Two additional specimens were recently collected by DROP from Curaçao that closely match the holotype of *P*. *paradoxus* in having a forked tail and vomerine teeth, but differ in some regards from the specimens from the Gulf of Mexico and Panama. At present, we consider the specimens from Curaçao and the single specimen from Venezuela to be conspecific with the holotype. Consequently, the characters shared amongst these specimens are those used in the key and comparison table (Table 2). The specimens from the Gulf of Mexico and Panama likely represent one, or possibly two, additional species, but additional material is needed to clarify their status.

**Table 2:** Comparison of diagnostic characters for *Palatogobius*.

	*P*. *incendius*	*P*. *grandoculus*	*P*. *paradoxus*
D2 (total)	I,14–16	I,17–18	I,18–20
A (total)	I,14–16	I,18–19	I,19–21
P	18–20	21	usually 19 (18–22)
Pelvic frenum	present (often damaged)	absent	absent
Body Scales	absent	present	present
Vomerine teeth	no	no	yes
Caudal shape	variable	lanceolate	forked*
Pore C	absent	singular	singular
Pore D	paired	singular	singular
Eye Diameter (%SL)	7.0–9.0	9.7–11.3	7.4–8.7
Behavior	large schools	singular or small groups	singular or small groups

*see Discussion on variation in this character

### Lionfish preying on undescribed species

Our submersible observations reveal that *Palatogobius incendius* may be especially susceptible to lionfish predation because of their hovering and schooling behavior. The schools are apparently easy to herd into corners by the slow-moving lionfish, and when startled, instead of taking shelter in the reef substrate, the gobies typically scatter or split into smaller schools, only to reform again as a large group. In these regards, *P*. *incendius* occupies a similar niche on deep reefs that *Coryphopterus personatus* and *C*. *hyalinus* occupy on shallow reefs. Both species of *Coryphopterus* are tiny, brightly colored, shallow-bodied species that form large schools that hover above the reef, and both are frequently the most abundant goby found in lionfish guts [14, 22, 28].

Other deep-reef fishes may avoid predation by being more tightly associated with the substrate. For example, *Lipogramma* species can be seen hovering above the reef feeding on plankton, but when startled individuals dart into piles of rubble or crevices in the rock wall. Other deep-reef gobies, including *Palatogobius grandoculus* and some species of *Varicus*, readily shelter in burrows in the sediment when startled. However, lionfish may still be significant threats to deep-reef species that shelter in the substrate, particularly if lionfish are targeting as prime hunting grounds specific habitat types where fishes are likely to be found. Indeed, as would be expected, lionfish are more commonly seen around wall and cave habitats and rocky reef slopes than they are on less complex habitats that harbor fewer deep-reef fishes (authors’ personal observations). Lionfish are especially abundant around man-made structures at mesophotic and deeper depths, such as shipwrecks, anchors, and garbage piles. For example, near a pile of steel debris ~4 m across at a depth of 67 m off Dominica we observed at least 23 lionfish. On a later dive off Dominica, we observed a series of ropes and chains attached to a sunken buoy at 148 m depth on a silty flat with no other nearby benthic structure. The debris was habitat for several deep-reef fishes, including an undescribed seabass in the genus *Baldwinella*, and was surrounded by approximately 12 lionfish.

It is unclear to what extent the lionfish observed on deep reefs are feeding exclusively at depth, or alternatively, whether they undergo vertical movements such that a significant portion of their diet includes shallow species. Gut-content information from lionfish from deep reefs would provide a better understanding of the percentage of deep-reef species in lionfish diets, as well as the number of species (including undescribed species) that are being consumed. There are no published records on the gut contents of lionfish from deep reefs, and to date, *P*. *incendius* has not been found in the few lionfish guts we have collected from deep reefs (many of which have been empty). Given that *P*. *incendius* has yet to be observed in a lionfish gut, it is conceivable that lionfish might not regularly feed on this species, and the observation recorded here might instead be a chance event, possibly induced by the bright lights of the submersible stunning the gobies and making them easier prey. This is unlikely for several reasons: (i) as discussed above, lionfish readily feed on similarly sized, schooling gobies like *Coryphopterus* spp. on shallow reefs; (ii) lionfish are especially active hunters in deep, low-light conditions [32,35], regardless of whether they are aided by artificial lights; (iii) unlike some other species of deep-reef fishes, schooling gobies do not appear to be stunned by the submersible lights, and will readily move in groups to avoid being collected by the submersible, or in response to feeding attempts of lionfish. Nevertheless, until we gather more data from deep-reef lionfish guts, our inferences about the true predation levels will be limited. While some species of shallow-reef gobies have recently been listed as vulnerable or threatened by the IUCN due to risks posed by lionfish, at present we do not advocate such action for *P*. *incendius*. The species was abundant at nearly every location we have explored with the submersible, and the density of lionfish on reefs at 100–200 m appears to be less than that of shallow reefs. We conclude that lionfish likely pose no immediate extinction threat for *P*. *incendius*.

Lionfish are difficult to capture with the *Curasub*, as they appear unaffected by the quinaldine anaesthetic used by the submersible, and spearing multiple individuals from the sub on a single dive is feasible but difficult. We are currently experimenting with prototypes of lionfish-specific traps that can be recovered with the *Curasub*, but at present the best method for capturing lionfish at depth for gut content studies is with pole-spears by divers using closed-circuit rebreathers. Such divers, however, are limited to about 150 m, decompression times can be extensive, and lionfish are known to extend much deeper. Until we can more efficiently sample lionfish at depth we will have an incomplete understanding of the severity of the threat lionfish pose to deep-reef communities. Fortunately, manned submersibles like the *Curasub* represent useful tools that enable access to and study of the diverse cryptobenthic deep-reef fish community, ultimately enabling formal taxonomic descriptions of unknown biodiversity before species become threatened or worse.

### Key to the species of *Palatogobius*

1a. Second dorsal fin I,14–16; anal fin I,14–17; body without scales except for occasionally 2 to 3 large cycloid scales on caudal-fin base; in life, lateral midline of body with bright, yellow/orange stripe that broadens on and extends to tip of caudal fin (Figs 1 and 2); vomerine teeth absent; interorbital pore C absent; interorbital pores D paired…. *Palatogobius incendius*

1b. Second dorsal fin I,17–20; anal fin I,18–21; body with scales, at least on posterior trunk; side of body uniformly light yellow, pink, or pale, occasionally with an iridescent blue horizontal strip above lateral midline (Figs 7 and 8); vomerine teeth present or absent; interorbital pore C present; interorbital pore D singular………2

2a. Pectoral rays 21; vomerine teeth absent; caudal fin lanceolate; scales on body extending anteriorly to beneath first dorsal fin…… *Palatogobius grandoculus* (Fig 8)

2b. Pectoral rays 18–20; vomerine teeth present; caudal fin forked; scales on body not extending anteriorly beyond vertical through middle of second dorsal fin…. *Palatogobius paradoxus* (Fig 7)

## Supporting information

S1 File A key to the genera of the *Microgobius* group of the Gobiosomatini (Gobiidae). (DOCX)

S1 Table Appendix. Contains GenBank numbers and GenSeq information for new sequences generated in this study.
(XLSX)
