# Supplementary material for: A new mesophotic goby, Palatogobius incendius (Teleostei: Gobiidae), and the first record of invasive lionfish preying on undescribed biodiversity
Source: PLoS One. 2017 May 25;12(5):e0177179. doi: 10.1371/journal.pone.0177179 (PMC5444649; doi:10.1371/journal.pone.0177179)
Supplement: S1 File — (DOCX) [file pone.0177179.s001.docx]

**Key to the western Atlantic species of the *Microgobius* Group (Gobiidae: Gobiosomatini)**

Note: Characters used in the generic key do not necessarily apply to Pacific members of the genera.

1a) Second dorsal and anal fins confluent with caudal fin; eyes extremely small... *Akko* (*A. dionea*)

1b) Second dorsal and anal fins distinctly separate from caudal fin; eyes moderate to large...2

2a) Preopercular pores absent…….3

2b) Two or three preopercular pores present……4

3a) Anal-fin rays usually I,12; head dorsoventrally depressed; body shape robust, body at origin of first dorsal fin distinctly deeper than at caudal peduncle…..*Parrella* (*P. macropteryx*)

3b) Anal-fin rays I,14 or greater; head round or slightly laterally compressed; body elongate, depth nearly uniform from head to caudal peduncle…*Palatogobius*

4a) Top of head naked; second dorsal-fin rays typically I,14-17........................................ *Microgobius* (see [52] for species key)

4b) Top of head scaled; second dorsal-fin rays typically I,11-13, rarely I,14...................................... 4

5a) Dorsal spines 1-4 greatly elongate, longest spine reaching to or extending beyond posterior margin of second dorsal fin when depressed; body with distinct broad yellow band along lateral midline (white in preservation); pelvic frenum with smooth posterior margin, frenum lacking thickened collagenous supports ................... *Antilligobius* (*A. nikkiae*)

5b) Dorsal spines 1-4 may or may not be elongate, but never extending to posterior margin of second dorsal fin when depressed; body not pigmented as stated above; pelvic frenum with scalloped margin, frenum supported with collagenous thickenings.................... *Bollmannia* (see [52] for species key)
